# Programmed death‐ligand 1 expression on CD22‐specific chimeric antigen receptor‐modified T cells weakens antitumor potential

**DOI:** 10.1002/mco2.140

**Published:** 2022-05-29

**Authors:** Jie Liu, Fengjuan Zhang, Jian Yu, Qi Zhao

**Affiliations:** ^1^ Department of Biochemistry School of Medicine Southern University of Science and Technology Shenzhen 518055 China; ^2^ Cancer Centre Faculty of Health Sciences University of Macau Taipa 999078 China; ^3^ MoE Frontiers Science Center for Precision Oncology University of Macau Taipa 999078 China; ^4^ School of Engineering Medicine Beihang University Beijing 100083 China

**Keywords:** CAR‐T, CD22, immune checkpoint, PD‐L1

## Abstract

The molecules of programmed cell death protein‐1 (PD‐1) and ligand‐1 (PD‐L1) become new therapeutic targets for cancer therapy. Although tumor‐expressed PD‐L1 molecule is frequently dispensable for checkpoint blockade in some cancer patients, recent studies suggest that T cell‐expressed PD‐L1 molecule might play a crucial role in antitumor immunity. Here, to investigate CD22 chimeric antigen receptor (CAR)‐T cell therapy, we have generated the different CD22 CAR‐T constructs. We noticed that tumor cells induced PD‐L1 expression on the surface of CD22 CAR‐T cells. The induced PD‐L1 might limit immunogenic responses of CAR‐T cells. T cell‐expressed PD‐L1 leads to a suppressive signal by PD‐1/PD‐L1 engagement of CD22 CAR‐T cells. Meanwhile, PD‐L1 suppresses CD22 CAR‐T cell differentiation into memory T cells and negatively affected secretions of several essential cytokines, such as interleukin‐2 (IL‐2) and tumor necrosis factor (TNF)‐α. We further observed that anti‐PD‐L1 monoclonal antibodies rescued cytokine secretion of CD22 CAR‐T cells rather than anti‐PD‐1 monoclonal antibodies. Our current studies provide a potential mechanism to understand the functions and roles of T cell‐expressed PD‐L1 in tumor microenvironment. These results will encourage the physicians to re‐recognize the important roles of PD‐L1 in cancer immunotherapy studies and provide the helpful guidance for clinical operation of PD‐L1 inhibition drugs.

## Graphical abstract

Engagement of CAR‐T cells through PD‐1/PD‐L1 ligation. We speculate that PD‐L1‐expressed CAR‐ T cells lead to a suppressive signal by PD‐1/PD‐L1 engagement. From there, T cell‐associated PD‐L1 is a major therapeutic target of immune checkpoint blockade.

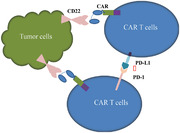

## 1 INTRODUCTION

The molecules of programmed death protein‐1 (PD‐1) and its ligand‐1 (PD‐L1) are important for immunosuppression in tumor microenvironment (TME).^1^ They represent the most crucial immune checkpoint molecules in regulation of T‐cell function.^2^, ^3^ Overexpression of PD‐1 was observed on chimeric antigen receptor (CAR)‐T cells against CD19 after they were infused into B‐cell malignancant patients.^4^ Several studies provide evidence that anti‐PD‐1/PD‐L1 inhibitors may augment the activities of CD19 CAR‐T cells.^5^, ^6^ In clinic, antibodies targeting PD‑1/PD‐L1 axis result in satisfied tumor regression in parts of cancer patients. Their results demonstrated that anti‐PD‐1 monoclonal antibody, pembrolizumab, effectively improved therapy of CD19 CAR‐T cells in some diffuse large B‐cell lymphoma patients.^7^, ^8^ The combination therapy with anti‐CD19 CAR‐T and PD‐1/PD‐L1 blockade has shown synergetic effectiveness in preclinical and clinical studies of hematologic tumors.^9^ However, it is interesting that PD‐1 knockdown in CD19 CAR‐T cells, which mimics long‐lasting PD‐1 blockade, fails to enhance the cytotoxicity in vitro or in vivo tumor models.^10^ This raises the question whether PD‐1 may not be the direct regulator in CAR‐T cells by PD‐1/PD‐L1 inhibitors. Therefore, it is necessary to exploit the unknown action mechanism of PD‐1/PD‐L1 inhibitors during CAR‐T cell therapy.

Beside tumor cells, PD‐L1 can be constitutively expressed by a variety of host immune cells.^11^ Increasing evidence suggests that PD‐L1 on host immune cells, rather than tumor cells, involves in tumor immune evasion in certain cancer patients that have negative/weak PD‐L1 expression in tumors.^12^, ^13^ Recently, Diskin and colleagues described that T cell‐expressed PD‐L1 might play a crucial role in antitumor immunity.^14^ They concluded that PD‐L1 was highly expressed by tumor‐infiltrating T (TIL) cells in TME. T cell‐expressed PD‐L1 suppressed tumor‐associated macrophages and TIL cells in a pancreatic tumor model. These findings imply that T cell‐expressed PD‐L1 is crucial for tumor regression.

As a B cell maturation marker, CD22 is a pan‐B antigen from early progenitor B cells to mature B cells and restricted on plasma cells.^15^ It has been well‐studied as a promising target for B‐cell malignancies using different anti‐CD22 therapies, such as monoclonal antibodies and CAR‐T cells.^16^ Some clinical results demonstrate that the CD22 CAR‐T cell treatment is active for CD22‐resistant acute B‐cell leukemias.^17^, ^18^ Although CD22 CAR‐T cells show promising, most patients relapse after they receive CD22 CAR‐T cell infusion. Similar to CD19 CAR‐T cells, a degree of relapse was seen in CD22 CAR‐T cell infusion.^19^ In this study, we have generated different CD22 CAR‐T constructs based on the anti‐CD22 monoclonal antibody m971.^19^ Using these CD22 CARs, we attempted to understand the potential involvement of PD‐1/PD‐L1 signal during anti‐CD22 CAR‐T cell therapy.

## 2 RESULTS

### 2.1 Generation of human T cells expressing anti‐CD22 CARs and PD‐1 extracellular domain

**FIGURE 1:**
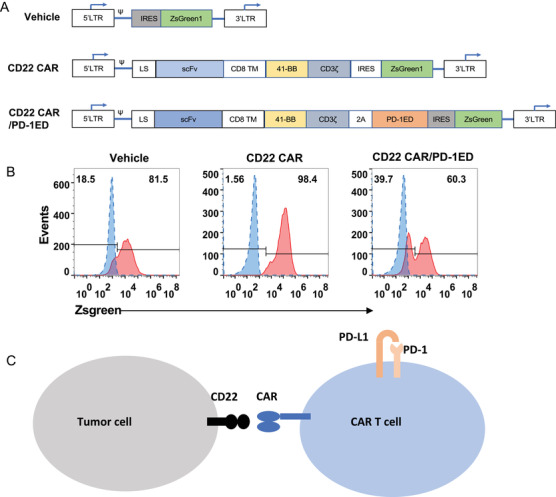
Generation of CD22 chimeric antigen receptor (CAR)‐T cells. (A) Schematic diagram of the different CAR constructs. The CD22 CAR contains CD8 leader sequence (LS), an anti‐CD22 scFv, a CD8 transmembrane domain (TM) sequences, the the CD3 zeta, and 4‐1BB sequence. For CD22 CAR/ programmed cell death protein‐1 (PD‐1)ED, the extracellular domain of the PD‐1 was linked by a P2A sequence to the anti‐CD22 CAR vector. (B) Transfection percentages of vehicle, CD22 CAR, and CD22 CAR/PD‐1ED in T cells analyzed by flow cytometry. Red color represents lentiviral transfected T cells. Blue color represents un‐transfected T cells. (C) Scheme diagram for PD‐1/programmed cell death ligand‐1 (PD‐L1) ligation in CD22 CAR‐T cells

We generated a CD22 CAR‐T construct containing the anti‐CD22 antibody scFv, the human CD8 transmembrane domain, the 4‐1BB (CD137) intracellular domains, and the CD3 zeta sequence (Figure 1A). Meanwhile, we generated a CD22 CAR/PD‐1ED construct in which the CD22 CAR was coupled by a P2A element to the PD‐1 extracellular domain (ED) containing a CD28 transmembrane domain. The lentiviruses were produced in HEK293T cells with a three‐plasmid package. The titer of purified virus is estimated as 5 × 10^8^ TU/ml with the measurement by flow cytometric analysis. Human peripheral blood monoclonal cells (PBMCs) were isolated on day 0 and transfected with the lentiviruses on day 1. After activation with the CD3/CD28 beads, PBMCs were growth for 2 weeks. After lentiviral transduction, the expression percentages of both CAR and vehicle exhibited 60%–98.4% in human T cells (Figure 1B). CD22 CAR‐T cells had similar patterns with vehicle in either CD4/CD8 ratios or expansion rates. The expanded CAR‐T cells presented the high viability (above 95%). Meanwhile, to investigate PD‐1/PD‐L1 ligation of CD22 CAR‐T cells, we established the CD22 CAR/PD‐1ED construct that co‐expressed the PD‐1 ED with CD22 CAR (Figure 1C). When co‐transfected PD‐1 ED with CD22 CAR, we noticed that PD‐1 ED expression was obvious on the cell surface of CD22 CAR/PD‐1ED T cells with detection of either anti‐PD‐1 antibody or human PD‐L1‐hFc protein using flow cytometry (Figure S1A). The mRNA expression of the PD‐1intracellular domain and ED in CD22 CAR‐T cells was determined by quantitative real‐time polymerase chain reaction (q‐PCR), respectively (Figure S1B).

### 2.2 Surface expression of CD22 and PD‐L1 in different tumor cell lines

**FIGURE 2:**
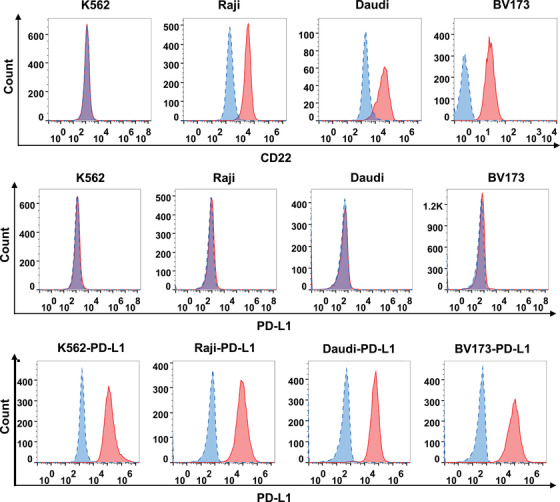
Expressions of CD22 and programmed cell death ligand‐1 (PD‐L1) on different tumor cells lines. Immunostaining was performed with anti‐human CD22 and anti‐human PD‐L1 antibodies. Red color represents antibody staining. Blue color represents nonantibody staining

We measured the expression levels of CD22 and PD‐L1 on different tumor cell lines (Raji, Daudi, BV173, and K562) using flow cytometry (Figure 2). For CD22 expression, Daudi and Raji showed the high expression as well as BV173 showed moderate expression in BV173. K562 cell line was CD22 negative. For PD‐L1 expression, although some studies demonstrated that PD‐L1 could be highly induced by interferon (IFN)‐γ‐treated tumor cells, we observed that PD‐L1 expression was relatively weak in these tumor cell lines with IFN‐γ treatment. To enhance the expression level of PD‐L1 in tumor cells, the lentiviruses carrying PD‐L1 genes were used to transfect K562, Raji, Daudi, and BV173 cells. Flow cytometric analysis indicated that all cell lines highly expressed PD‐L1 with anti‐PD‐L1 antibody staining.

### 2.3 Tumor cell‐induced expression of PD‐L1 on CD22 CAR‐T cells

**FIGURE 3:**
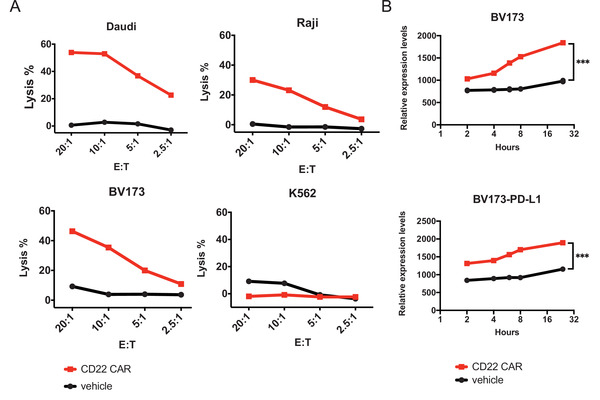
In vitro cytotoxicity and programmed cell death ligand‐1 (PD‐L1) expression of CD22 chimeric antigen receptor (CAR)‐T cells. (A) Cytotoxicity of CD22 chimeric antigen receptor (CAR)‐T cells and vehicle to tumor cells (Daudi, Raji, BV173, and K562). (B) Flow cytometric analysis of PD‐L1expression on the surface of CD22 CAR‐T cells that were co‐cultured with BV173 or PD‐L1‐transfected BV‐173 cell lines at different time points. Mean fluorescent intensity (MFI) ratio represents a ratio of MFI over unstained samples

The cytotoxic activities of CD22 CAR‐T cells against CD22‐positive and CD22‐negative cells were analyzed using the calcium‐AM release assay. The cytotoxic activities of CD22 CAR‐T cells were tested against three CD22‐positive tumor cell lines (Raji, Daudi, BV173) and one CD22‐negative cell line (K562) (Figure 3A). Target tumor cells, which were prestained with Calciem‐AM dye, were treated with CAR‐T cells with four effector‐to‐target (E:T) ratios for 4 h, including 2.5:1, 5:1, 10:1, and 20:1. All of CD22‐positive tumor cell lines but not CD22‐negative cells efficiently responded to CD22 CAR‐T cells. CD22 CAR‐T cells exhibited specific killing activity against Daudi cells (∼55%), Raji cells (∼30%), and BV173 cells (45%) at the 20:1E/T ratio, while no obvious killing was observed on CD22‐negative K562 cells. Vehicle T cells did not show obvious cytotoxicity at all E:T ratios. The cytotoxicity ability of CD22 CAR‐T cells depends on the E/T ratio increase but not the expression level of CD22 on the surface of target tumor cells. This may be due to the status of tumor cells and their sensitivity to CAR‐T cells.

After 24 h, CD22 CAR‐T cells were collected to measure the PD‐L1 expression on the cell surface. PD‐L1 was significantly upregulated on CD22 CAR‐T cells when they were co‐incubated with tumor cells at different time points. Interestingly, as shown in flow cytometric analysis (Figure 3B and Figure S2), PD‐L1 expression was continuously increased (*p *< 0.001). Furthermore, CD22 CAR‐T cells were incubated with either BV173 or BV173 PD‐L1 cell lines. We observed that PD‐L1 expressed by CD22 CAR‐T cells is irrespective of PD‐L1 expression in target tumor cells. The same tendency was observed when CD22 CAR‐T cells were incubated with Raji cells. The culture supernatant was harvested from tumor cells and was used to induce the expression of PD‐L1 on CD22 CAR‐T cells. No detectable expression was observed. Meanwhile, we attempted to induce the expression of PD‐L1 on PBMCs when they were co‐cultured with target cells. PD‐L1 was only slightly expressed in PBMCs. All results demonstrate that PD‐L1 induction on the CD22 CAR‐T cells is specifically associated with tumor cell stimulation.

### 2.4 Engagement of PD‐L1 weakens secretion of essential inflammatory cytokines

**FIGURE 4:**
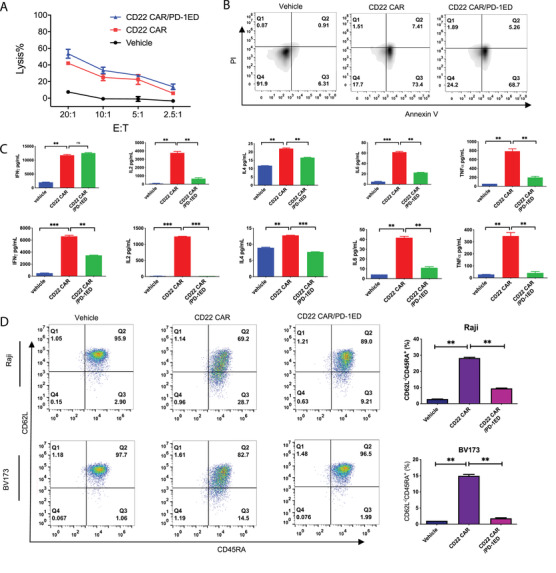
Programmed cell death ligand‐1 (PD‐L1) ligation decreases cytokine secretion and effector memory of CD22 chimeric antigen receptor (CAR)‐T cells. (A) Comparison of CD22 CAR, CD22 CAR/PD‐1ED, and vehicle killing Raji cells at different E:T ratios. (B) Apoptosis in target Raji cells induced by the different CD22 CAR‐T cells at E:T ratios of 10:1 for 4 h. Target tumor cells were stained with propidium iodide (PI) and fluorescent Annexin V. (C) Cytokine production in CD22 CAR‐T cells. CAR‐T cells were added into the 96‐well plates containing either Raji or BV173 cells at a 10:1 ratio for 24 h. (D) Effector memory cells of CD22 CAR‐T cells. The killing abilities of CAR‐T cells were tested against Raji and BV173 cells at a 10:1 E:T ratio for 24 h. Two T cell markers (CD62L and CD45RA) were detected with anti‐CD62L and anti‐CD45RA antibodies, respectively. The concentrations of cytokines, apoptosis, and cell surface markers were measured using flow cytometry

Recent evidence indicates that PD‐L1 expressed by TIL cells potentially dampens antitumor functions of effector T cells in TME. PD‐L1 decreases expressions of cytotoxic effector molecule (granzyme B) and secretions of inflammatory cytokine (TNF and IFN‐γ).^14^ However, the roles of T‐cell expressed‐PD‐L1 have not yet been clearly elucidated. In in vitro results, PD‐1/PD‐L1 engagement did not obviously change the cytotoxic and apoptotic abilities of CD22 CAR‐T cells against Raji tumor cells (Figure 4A,B). However, secretions of key cytokines (IFN‐γ, IL‐2, IL‐4, IL‐6, and TNF‐α) were significantly decreased in CD22 CAR/PD‐1ED T cells (Figure 4C). To determine T cell differentiation, the effector markers of CD8^+^ T cells were measured after CD22 CAR‐T cells were incubated with Raji or BV173 cells. Compared with CD22 CAR‐T cells, we observed that exhibited the proportion of CD62L^–^CD45RA^+^ effector memory CD8^+ ^T cells decreased in CD22 CAR/PD‐1ED T cells (Figure 4D). The decreased proportion of CD45RA^+^CD62L^– ^cells may reflect to weak the potential cytotoxic abilities of CD22 CAR/PD‐1ED T cells.

### 2.5 Anti‐PD‐L1 antibody rescued essential cytokine secretion

**FIGURE 5:**
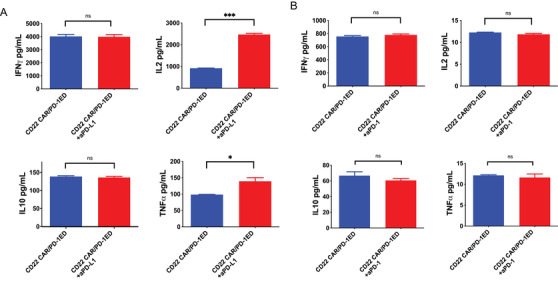
Anti‐ programmed cell death ligand‐1 (PD‐L1) antibody treatment rescues cytokine secretion from CD22 chimeric antigen receptor (CAR) T cells. (A) Cytokine secretions of CD22 CAR/PD‐1ED with the treatment of anti‐PD‐L1 antibodies at the concentration of 10 μg/ml. (B) Cytokine secretions of CD22 CAR/PD‐1ED with the treatment of or anti‐PD‐1 antibodies at the concentration of 10μg/ml. Effector T cells were incubated with target BV173 cells at a 10:1 ratio for 24 h. The cytokine levels were determined using flow cytometry

To improve cytokine secretions, CD22 CAR‐T cells were treated with anti‐PD‐1 and anti‐PD‐L1 monoclonal antibodies, respectively. Either CD22 CAR‐T or CD22 CAR/PD‐1ED cells were incubated with BV173 cells at the 10:1 ratio for 24 h. CAR‐T cells were treated with either anti‐PD‐1 or anti‐PD‐L1 monoclonal antibodies at the concentration of 10 μg/ml for 24 h. The cytokine levels in supernatants were determined by flow cytometry. We noticed that the anti‐PD‐L1 antibody treatment recovered the productions of IL‐2 and TNF‐α (Figure 5A). However, anti‐PD‐1 antibody did not show the same effect (Figure 5B). Above results suggest that the anti‐PD‐L1 antibodies potentially improve the effects of CD22 CAR‐T cells by suppressing the PD‐L1‐induced inhibitory effects. This observation seems to provide an explanation why some PD‐L1 negative patients respond to anti‐PD‐L1 treatment.

## 3 DISCUSSION

There has been great progress in CAR‐T cells in clinical therapy of hematological cancers. Recent studies suggest that immune checkpoint molecules can weaken the therapeutic effects of CAR‐T cells. Novel immune checkpoint inhibitors targeting immune checkpoint have shown success in controlling tumor progress. The PD‐1/PD‐L1 molecules are typical immune checkpoints. the United States Food and Drug Administration (US‐FDA) has approved two PD‐1 blockade drugs for treating melanoma in 2014 and nonsmall cell lung cancer (NSCLC) in 2015, respectively.

Here, we generated a CD22‐specific CAR‐T construct based on the scFv of anti‐CD22 antibody m971. We noticed that PD‐L1 expression was upregulated on CD22 CAR‐T cells. We hypothesize that PD‐L1 expression on CAR‐T cells is triggered by tumor cell direct interaction. To investigate whether tumor‐induced PD‐L1 affects the functions of CD22 CAR‐T cells, we further generated a CD22 CAR/PD‐1ED construct expressing the PD‐1 ED that can lead to engagement of CAR‐T cells through PD‐1‐PD‐L1 ligation. Compared with CD22 CAR, CD22 CAR/PD‐1ED does not affect T cell proliferation and CD4/CD8 population. Most T cells mediate cytolytic effector functions by inducing target cell apoptosis. We observed that the PD‐L1 engagement did not change the apoptosis rates of target cells in the short‐term culture with CAR‐T cells. However, the PD‐L1 engagement alters a small number of T cells differentiate into memory T cells. Secretions of IL‐2 and TNF‐α are significantly decreased with PD‐L1 expression on CD22 CAR‐T cells. The engagement may therefore weak the long‐term therapeutic effects of CAR‐T cells. This may give a reason why the CD22 CAR‐T cell activities are limited by preventing long‐term memory T cell differentiation.

Results of preclinical and clinical studies have demonstrated that combination of CAR‐T cells with PD‐1/PD‐L1 blockade can be beneficial for CAR T cell activity.^20^, ^21^ To investigate whether blockade of immune checkpoint inhibitors can improve the functional activities of CD22 CAR‐T cells and relieve PD‐L1 suppression, CD22 CAR‐T cells were treated with anti‐PD‐1 and anti‐PD‐L1 monoclonal antibodies, separately. We observed that secretions of two essential cytokines, including IL‐2 and TNF‐α, were restored in CD22 CAR/PD‐ED T cells with anti‐PD‐L1 antibody treatment. In contrast, the anti‐PD‐1 antibody failed to improve cytokine status in the CAR/PD‐1ED treatment group. One potential explanation is that ligands are more effectively blocked than receptors.^22^ However, further studies are needed to determine the unclear mechanism. This observation indicates that administration of the PD‐L1 antibody rather than the PD‐1 antibody blockade may be sufficient to improve the therapeutic outcome of CD22 CAR‐T cell therapy.

Elucidation of PD‐L1 expressed by host immune cells is important for cancer research scientists and physicians who need to understand how PD‐1/PD‐L1 blockade benefits patients. Our current work may provide the observation that T cell–expressed PD‐L1 should be comprehensively investigated in immunotherapy. We speculate that PD‐L1 expressed CAR‐T cells lead to a suppressive signal by PD‐1/PD‐L1 engagement. From there, T cell‐expressed PD‐L1 is a major therapeutic target of immune checkpoint blockade. In another study, we reported that the PD‐L1 was observed to express on B7‐H3 CAR T cells as well as PD‐L1 blockade might effectively increase the killing ability of CAR‐T cells against NSCLC cells.^23^ Thus, we can conclude that the PD‐L1 expressed on antigen‐activated CAR‐T cells is highly associated with cytokine secretion and has negative effects on antitumor efficacy of CAR‐T cells. These results encourage our further investigation of PD‐L1 functions in CAR‐T cells. However, PD‐L1 engagement and signals have not yet been defined for antitumor immunity. Future studies are necessary to investigate whether T cell‐expressed PD‐L1 affects the in vivo anti‐tumor activities of CD22 CAR‐T cells in primary cell or patient‐derived xenografts.

## 4 MATERIALS AND METHODS

### 4.1 Cell lines and culture

Several cell lines, including HEK293T, K562 (Chronic Myelogenous Leukemia), Raji (Burkitts Lymphoma), and Daudi (Burkitts Lymphoma), were gifted by the Shanghai Cell Bank, Chinese Academy of Sciences. The BV173 (chronic myelogenous leukemia) cell line was obtained from CLS Cell Line Service. Four PD‐L1^+^ cell lines (Raji‐PD‐L1, Daudi‐PD‐L1, K562‐PD‐L1, and BV173‐PD‐L1) were generated using the lentiviral vector carrying human PD‐L1. Human PBMCs were isolated from the healthy adult donors. Above cells were grown in completeRoswell Park Memorial Institute (RPMI)‐1640 (GIBCO) containing 10% fetal bovine serum (FBS) (GIBCO), 1x penicillin, and streptomycin at 37℃ with 5% CO_2_. For PBMC culture, 100 U/ml recombinant human IL‐2 (rhIL‐2) (CellGenix) is added into RPMI‐1640 medium.

### 4.2 Lentiviral production

The amino acid sequence of anti‐CD22 single chain variable fragment (scFv) was obtained from one anti‐CD22 monoclonal antibody, m971. The contruct of CD22 CAR was designed to include CD8 leader peptide, CD8 transmembrane domain sequence, anti‐CD22 scFv, the 4‐1BB intracellular domain, and the CD3ζ sequence. For CD22 CAR/PD‐1 ED, the ED sequence of the PD‐1 was fused to the CD28 transmembrane domains and linked by a P2A sequence to the CD22 CAR vector. All constructs were cloned into lentivirus backbone plasmids. Lentiviruses carrying the CARs were generated with transfection of backbone plasmid and two packaging plasmids, pMD2.0G and PsPAX2, in HEK293T cells as previously described.^24^ The culture supernatants were harvested and filtrated by 0.45 μm filters (Millipore), followed by concentration by ultra‐centrifugation at 28,000 rpm for 2 h. Titers were calculated by counting the infected HKE293T cells based on the ZsGreeen reporter using the flow cytometer.

### 4.3 T cell isolation and transduction

The buffy coat samples were prepared from the health adult donors. Human PBMCs were freshly isolated using Ficoll–Paque PLUS reagents following the manufacturer's procedures. CD56+ natural killer (NK) cells were removed by anti‐human CD56 beads (BD Biosciences). PBMCs were cultured in the RPMI‐1640 medium at the presence of rhIL‐2 and then activated by anti‐human CD28/CD3 beads at a 1:1 ratio. After 24 h, the lentiviruses were added at a 5:1 multiplicity of infection. After adding polybrene, the lentiviral transfection was performed using a centrifuge at 1000 rpm/min for 1 h at 30°C. The beads were removed by the magnetic stand after 7 days. All CAR‐T cells were maintained in complete RPMI‐1640 containing 10% FBS at the presence of rhIL‐2 until used in the assays.

### 4.4 Q‐PCR

Briefly, RNA was prepared from cell samples using Trizol reagent (Invitrogen) following the manufacturer's procedures. The deoxyribonucleic acid (DNA) was synthesized from 2.5 μg of RNA. Then, the q‐PCR was performed with Bio‐Rad CFX96 by using iTaq Universal SYBR Green (Bio‐Rad). Glyceraldehyde 3‐phosphate dehydrogenase (GAPDH) was used as normalizers. Six pairs of primers were designed to amplify the target fragments of GAPDH, intracellular domain of PD‐1, and ED of PD‐1. The primers sequences are as follows: GAPDH: 5′‐CACCGTCAAGGCTGAGAACG‐3′ and 5′‐CATGGTGGTGAAGACGC CA‐3′; PD‐1 Intra: 5′‐CAGCCGTGCCTGTGTTCT‐3′ and: 5′‐GGTGCCCATTCCGCTAGG‐3′; PD‐1 EM: 5′‐AACGGGCGTGACTTCCACAT‐3′ and: 5′‐ GGCACTTCTGCCCTTCTCTCT‐3′, and PD‐L1: 5′‐TATGGTGGTGCCGACTACAA‐3′ and 5′‐TGCTTGTCCAGATGACTTCG‐3′.

### 4.5 Flow cytometry

The expressions of CD22 and PD‐L1 on tumor cell lines or T cells were detected with fluorescein isothiocyanate (FITC) anti‐human CD22 antibody (BioLegend) and phycoerythrin (PE) anti‐human PD‐L1 antibody (BioLegend), respectively. The CD22 CAR on the cell surface was detected with CD22‐Fc protein (Sinobiological), followed by the PE anti‐human IgG antibody (Jackson ImmunoRes) as the secondary antibody. The expression of PD‐1ED on cell surface was stained with APC‐labeled anti‐human PD‐1 antibody (BioLegend) and human PD‐L1‐hFc protein (R&D Systems), respectively. Flow cytometry was performed using either BD Accuri C6 or Beckman CytoFLEX S.

### 4.6 Expression and purification of anti‐PD‐L1 IgG

The amino acid sequences of the anti‐PD‐L1 monoclonal antibody were obtained from atezolizumab. The synthesized DNA sequences were ligated with the human IgG1 constant region in the expression vector. To express antibodies in 293‐S cells, anti‐PD‐L1 IgG1 plasmids (1 μg/ml) and 40 μl of PEI transfection reagents (1 μg/ml) were mixed for 10 min at room temperature. Then the mixture was dorpwised to suspension 293‐S cells and growth in serum‐free expression medium in a CO2 shaker. After 4 days of posttransfection, the IgG proteins were purified by the Protein A resins from the culture supernatant.

### 4.7 Cytotoxicity assay

The T cell‐mediated cytotoxicity assays were performed by the Calcein‐AM releasing method as previously described.^24^ Briefly, total 1 × 10^6^ cells/ml of tumor cells were prestained with the dye Calcein‐AM at the concentration of 10 μmol/L at 37°C for 30 min. After washing, prestained tumor cells were treated with the different T cells in the 96‐well plates with different E:T ratios from 2.5:1 to 20:1. After 4 h, the supernatants were harvested. Mean fluorescence intensity was determibed at 495/515 nm using the PerkinElmer Multimode Reader. The killing percentages in the sample wells were calculated according to the formula:

$$\begin{array}{ccc} & & \text{Killing}\;\text{percentage}=\left(\;\mathrm{MF}I_{\text{sample}\;\text{lysis}}-\mathrm{MF}I_{\mathrm{spontaneous}\;\text{lysis}}\;\right)\;/\\ & & \;\left(\mathrm{MF}I_{\mathrm{maximum}\;\text{lysis}}-\mathrm{MF}I_{\mathrm{spontaneous}\;\text{lysis}}\right)\times 100\%. \end{array}$$

### 4.8 Assessment of secreted cytokines

To measure the cytokine secretions of CAR‐T cells, effector T cells were added to the plates containing target tumor cells at 10:1 E:T ratio for 24 h. The cytokine levels in the culture medium were counted with Human Th1/Th2 Cytokine Cytometric Bead Array (CBA) Kit II using the flow cytometer. 

### 4.9 Statistical analysis methods

The data are presented as mean ± SEM. The GraphPad Prism software was used for the statistical analysis. Significant values were determined with the two ways analysis of variance (ANOVA) or *t* tests and presented as: *p < 0.05, **p < 0.01, ***p < 0.001, and ****p < 0.0001.

## CONFLICT OF INTEREST

The authors declare that they have no conflict of interests.

## AUTHOR CONTRIBUTIONS

Jie Liu and Fengjuan Zhang contributed equally to the experiments. Jie Liu and Fengjuan Zhang executed experiments of CART. Qi Zhao supervised all aspects of the projects. Jian Yu and Qi Zhao co‐wrote the manuscript.

## ETHICS STATEMENT

The data collections from human participants have been agreed by the Human Ethics Committee of the University of Macau (approval number is BSERE17‐APP020‐FHS). The animal experimental studies have been agreed by the Animal Ethics Committee of the University of Macau (approval number is UMARE‐018‐2017).

## Supporting information

Supporting information

## Data Availability

The data included in this study are available upon request from the corresponding authors.
