# Supplementary material for: Programmed death‐ligand 1 expression on CD22‐specific chimeric antigen receptor‐modified T cells weakens antitumor potential
Source: MedComm (2020). 2022 May 29;3(2):e140. doi: 10.1002/mco2.140 (PMC9149589; doi:10.1002/mco2.140)
Supplement: Supplementary file 1 — Supporting information [file MCO2-3-e140-s001.docx]

# Programmed death-ligand 1 expression on CD22-specific chimeric antigen receptor-modified T cells weakens antitumor potential

Jie Liu^1#^, Fengjuan Zhang^2,3#^, Jian Yu^4,5*^, Qi Zhao^2,3*^

^1^Department of Biochemistry, School of Medicine, Southern University of Science and Technology, Shenzhen, China

^2^Cancer Centre, Faculty of Health Sciences, University of Macau, Taipa, Macau, China

## ^3^MoE Frontiers Science Center for Precision Oncology, University of Macau, Taipa, Macau, China

^4^School of Engineering Medicine, Beihang University, Beijing, China

^5^Beijing Advanced Innovation Center for Biomedical Engineering, Beihang University, Beijing, China


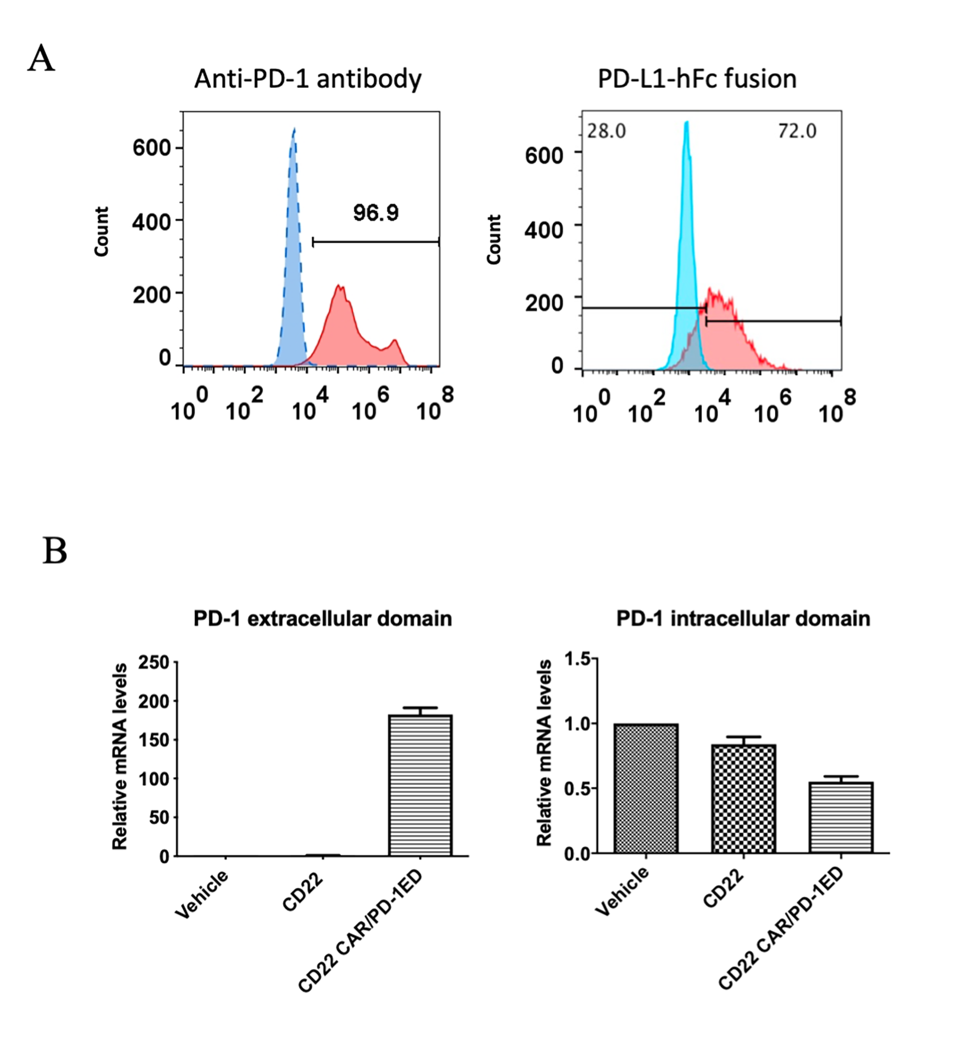


**Figure S1.** PD-1 expression in the CAR T cells. A. Expression of PD-1 extracellular domain in CD22 CAR/PD-1ED T cells after lentiviral transduction as assessed by anti-PD-1 antibody or human PD-L1-hFc fusion protein with flow cytometry. B. The relative mRNA level of PD-1 extracellular domain and intracellular domain in the different CAR-T cells compared to vehicle T cells as analyzed by qPCR.


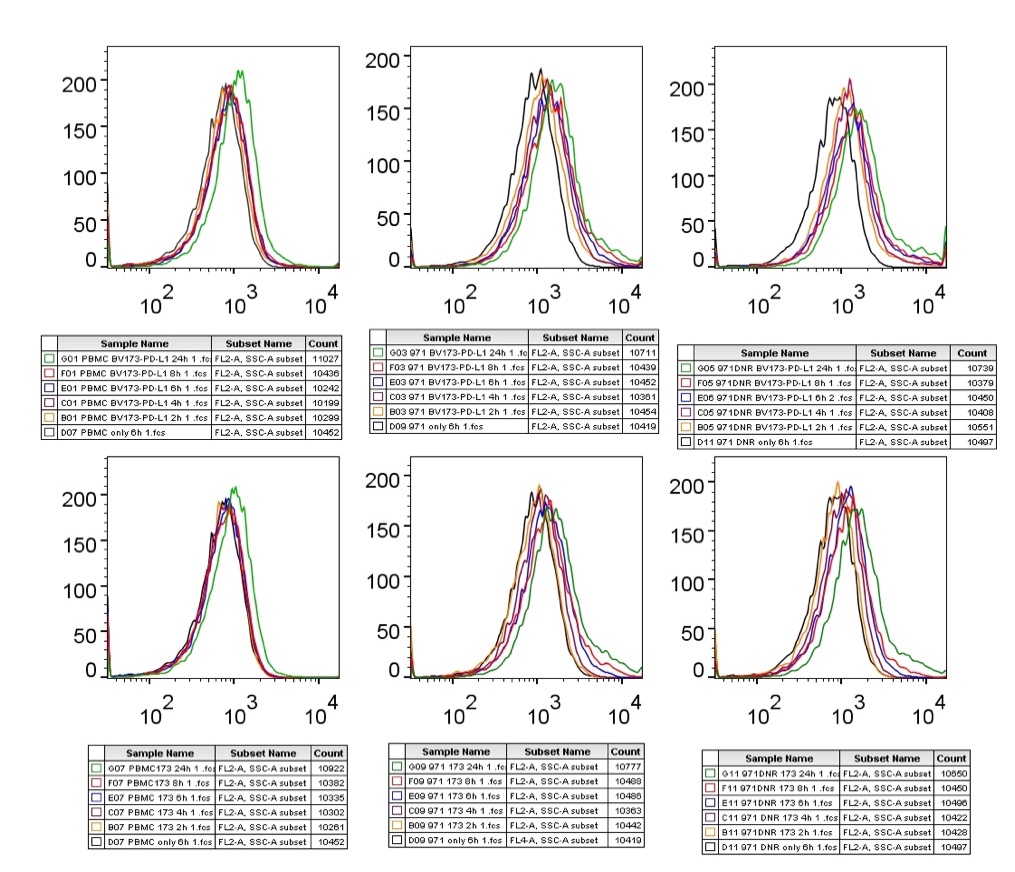


PD-L1-PE

Count

BV173

BV173

-PD-L1

Vehicle


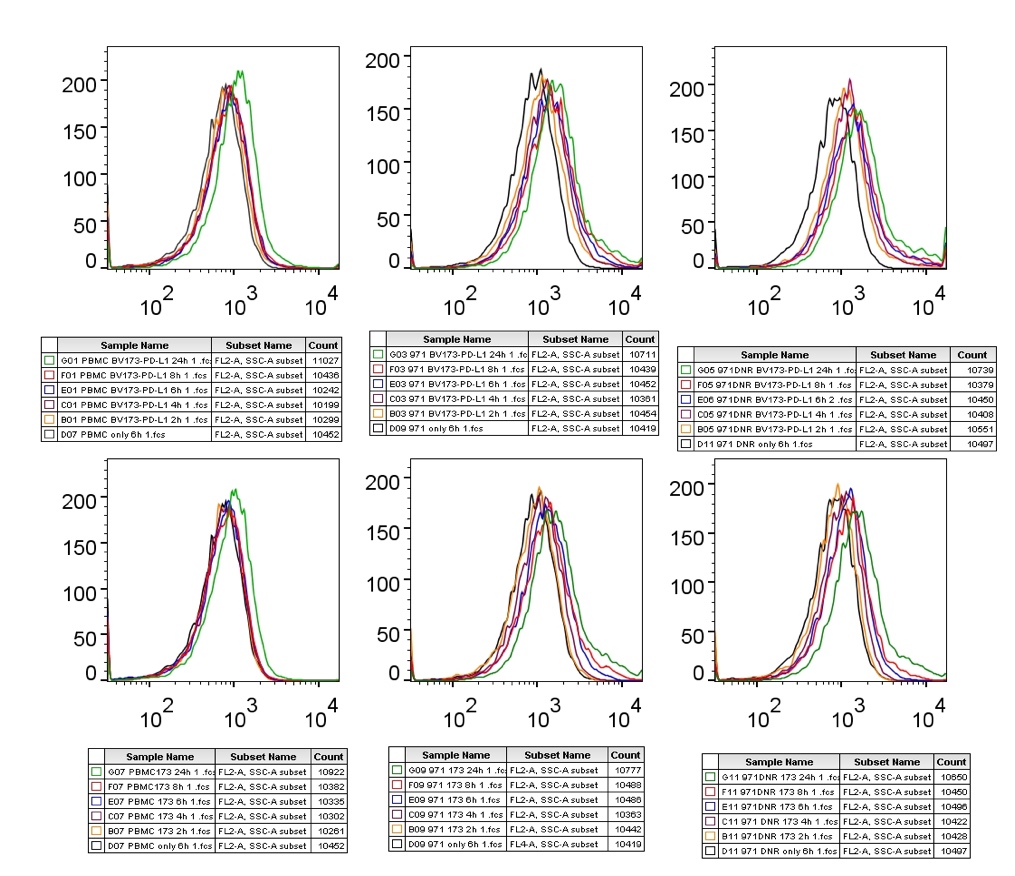


0h

2h

4h

6h

8h

24h

**Figure S2** Flow cytometry analysis of the PD-L1 expression on CD22 CAR T cells when co-cultured with BV173 or BV173-PD-L1. Representative results are shown for one experiment with three PBMC donor from three independent experiments.
